# A small-sized imidazole-derived ligand binds to the KRAS promoter G-quadruplex and inhibits cancer growth with enhanced immunomodulation

**DOI:** 10.1016/j.jbc.2025.110647

**Published:** 2025-08-28

**Authors:** Xiao-Dong Wang, Qian-Wen Nie, Ming-Hao Hu

**Affiliations:** Nation-Regional Engineering Lab for Synthetic Biology of Medicine, International Cancer Center, School of Pharmacy, Shenzhen University Medical School, Shenzhen, China

**Keywords:** KRAS G-quadruplex, wide-spectrum inhibitor, immunomodulation

## Abstract

KRAS overactivation is commonly present in a diversity of solid tumors. Recently, small-molecule inhibitors of the KRAS G12C mutation have been approved for clinical use, marking the end of the long era of KRAS as an “undruggable” target. However, new approaches to suppress a wide spectrum of KRAS abnormalities are still needed to be developed. G-quadruplex (G4) is located in the nuclease hypersensitive element (NHE) region of the promoter and controls KRAS expression. To date, only a few KRAS G4 ligands with coplanar aromatic scaffolds have been discovered. These compounds fall outside the “drug-like” chemical space and lack satisfactory selectivity between KRAS G4 and other DNAs. In this study, a series of drug-like, indolium-based analogs binding toward KRAS G4 were engineered, and BN1, derived from imidazole, was selected as the most potent ligand. BN1 effectively suppressed KRAS expression, thereby downregulating the MEK-ERK pathway and PD-L1 expression in tumor cells. *In vivo* experiments demonstrated that BN1 effectively reduced tumor burden while exhibiting immunostimulatory effects, including the increase of CD8^+^ IFNγ^+^ cells, the decrease of CD4^+^ Foxp3^+^ cells, and the regulation of cytokines. Collectively, it is the first time, to our knowledge, to report a KRAS G4-directed small-molecule ligand with antitumor efficacy related to enhanced immunomodulation.

Three RAS family proteins (KRAS, NRAS, and HRAS) are GTPases of 21 kDa, which cycle between the active GTP-bound and the inactive GDP-bound states, a process involving the action of RAS-guanine nucleotide exchange factors (RAS-GEFs) and RAS-GTPase-activating proteins (RAS-GAPs), respectively (1, 2, 3). RAS proteins are among the most frequent abnormalities in cancer, where KRAS mutations or amplifications are commonly present in solid tumors (4, 5). GTP binding to KRAS stimulates downstream signaling pathways, notably MAPK pathway and PI3K pathway, thereby initiating cell proliferation and differentiation. KRAS mutations in GTPase limit the hydrolyzation of GTP to GDP, and sustain the signaling activation, resulting in tumorigenesis and tumor progression (6, 7). KRAS also has multiple immunomodulatory roles, not only altering the behaviors of cancer cells, but also exerting effects on a diversity of cells in tumor microenvironment (TME), like immune cells, fibroblasts and endothelial cells (8). KRAS amplification contributes to numerous immunosuppressive implications, such as polarization of M1 to M2 macrophages and inhibition of CD8^+^ T cells *via* MHC-TCR (T cell receptor) signaling. In addition, KRAS enhances tumor infiltration of MDSCs (myeloid-derived suppressor cells) and Tregs (regulatory T cells) through several mechanisms (9, 10).

PD-L1, a representative immune checkpoint, is always aberrantly upregulated on cancer cells to facilitate the tumors to evade immune destruction. PD-L1/PD-1 signaling is of great importance in the regulation of TME, and antibodies blocking PD-L1 or PD-1 are reported to be promising in the treatment of several malignancies with KRAS mutation (11, 12). Clinical trials report that the PD-1 antibody is more beneficial to patients with NSCLC (non-small cell lung cancer) having KRAS mutation than other patients (13). Furthermore, molecular mechanisms of KRAS signaling on the upregulation of PD-L1 expression are clarified, involving the stabilization of PD-L1 mRNA *via* the AU-rich element-binding protein tristetraprolin (TTP) (14).

Despite the far-reaching consequences, KRAS has been considered ‘undruggable’ for several decades, due to the absence of an appropriate hydrophobic pocket on its surface to accommodate the small-molecule inhibitors (15, 16). Breakthroughs were made in 2021 and 2022, when Sotorasib and Adagrasib were approved by the FDA for the treatment of advanced-stage NSCLC patients with KRAS G12C mutation (17, 18). However, there are still some limitations for KRAS inhibitors in clinical use. First, they are only efficacious against the G12C mutant, leaving other types of mutants far from curative (19). Second, resistance of KRAS-directed drugs remains evitable, with tumor cell-autonomous as well as non-autonomous (for example, related to TME) mechanisms, which can be further categorized as primary or acquired resistance. The reasons for acquired resistance can be mutational escape of KRAS itself or KRAS mediators (20, 21). Therefore, new approaches capable of suppressing a wide spectrum of KRAS mutants, especially with antitumor immune responses, are still needed to be explored.

G-quadruplexes (G4s) are unique nucleic acid sequences folded into four-stranded secondary structures, frequently forming in guanine (G)-rich regions in telomeres and promoters of genes. It is believed that G4s play significant regulatory roles in a variety of diseases, mainly cancers and neurodegenerative disorders (22, 23). Recently, G4s in oncogene promoters are considered as powerful targets for cancer therapy, given their major implications in regulating transcription and translation. Small-molecule G4-targeted ligands, such as c-MYC, c-Kit, Bcl2, and KRAS binders, are designed or discovered to remarkably repress gene expression (24, 25).

As for KRAS oncogene, there are three potential promoter G4s located in the nuclease hypersensitive element (NHE) region, namely near, mid and far G4s. Near KRAS G4 (KRAS-32R) is a 32-nucleotide sequence about 115 bp upstream of TSS (transcription start site) with a parallel conformation (26, 27). A shorter 22-nucleotide sequence truncated from KRAS-32R with G16 T mutation (KRAS-22RT) is applied as a model for *in silico* studies, since it is more stable and has better resolved NMR peaks than wild-type KARS-22R (28). Several transcription factors are involved in KRAS expression by binding to near KRAS G4, including MAZ, hnRNP A1 and HMGB1 (29, 30). Small molecules are reported to compete KRAS G4 with nuclear proteins, resulting in the following KRAS inhibition (31, 32). For instance, a benzophenanthridine alkaloid destabilizes the interaction of hnRNP K with i-motif (mid) and stabilizes KRAS G4s (near, mid, and far), leading to KRAS downregulation and antitumor effects (33). Novel indoloquinoline compounds were identified to downregulate KRAS expression through selective stabilization of mid KRAS G4 (34). Nevertheless, few KRAS G4 ligands have been discovered to date (31, 32, 33, 35, 36), let alone their potentials and mechanisms on tumor therapy. Moreover, these developed ligands possess coplanar aromatic scaffolds with potentially poor solubility, high molecular weights as well as complicated multistep synthetic procedures, falling outside “drug-like” chemical space. In addition, such ligands have no satisfactory selectivity between KRAS G4 and other DNAs, which are even unable to differentiate among G4 topologies (parallel, antiparallel and hybrid), possibly leading to unexpected side effects if used as anticancer agents.

In this regard, we desire to discover novel drug-like G4 ligands that have good selectivity for KRAS G4 and good membrane permeability. There are some reported small-size G4 ligands (37, 38), but they only serve as fluorescent probes for G4 detection with little cytotoxicity on cancer cells, leaving a huge room for the development of new types of small-size ligands. Inspired by our previous studies on indolium-based scaffolds (39, 40, 41), in this study, we rationally designed and synthesized a new, focused library of small-size indolium-based analogs by attaching simple aromatic heterocycles, with structure–activity relationship (SAR) being discussed targeting KRAS G4 and BN1, derived from imidazole, being selected as the most promising ligand. Selectivity of BN1 for KRAS G4 than other types of G4s was proved by TO displacement, competitive fluorescence recovery, competitive FRET assays. The end-stacking mode of BN1 to both 3′- and 5′-ends of KRAS G4 was then verified by a ratio of 2:1. BN1 could inhibit the promoter activity of KRAS, as well as the transcription and translation of KRAS in the cells. RNA sequencing results indicated that multiple KRAS-directed antineoplastic and immunomodulatory pathways were involved, while BN1-induced suppressions of MEK-ERK and PD-L1 were detected in human triple-negative breast cancer (TNBC) MDA-MB-231 cells (KRAS G13D mutation) and mouse TNBC 4T1 cells (wild-type KRAS overexpression). Antitumor and immunostimulatory effects were finally decided in 4T1-bearing mice by the regulation of multiple types of T cells and cytokines. Notably, this study was the first to characterize a KRAS G4-targeted small-molecule ligand as a potent immunoactivator, both *in vitro* and *in vivo*.

## Results and discussion

### Design and synthesis of indolium-based analogs

**Figure 1:**
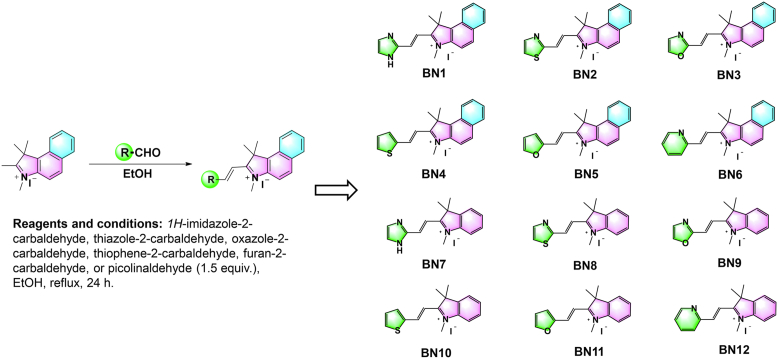
**Design and synthesis of indolium-based analogs (BN1–BN12), including synthesis routes (*left*) and chemical structures (*right*) of the compounds**.

All the designed ligands (BN1–BN12) had low molecular weights, and conformed to Lipinski’s “rule of five”, indicative of drug-like properties (Figure 1). All the ligands were synthesized in a single step through Knoevenagel condensation of commercially available materials. All the final products could be acquired as pure solids by filtration without further purification.

### Screen of indolium-based analogs

**Figure 2:**
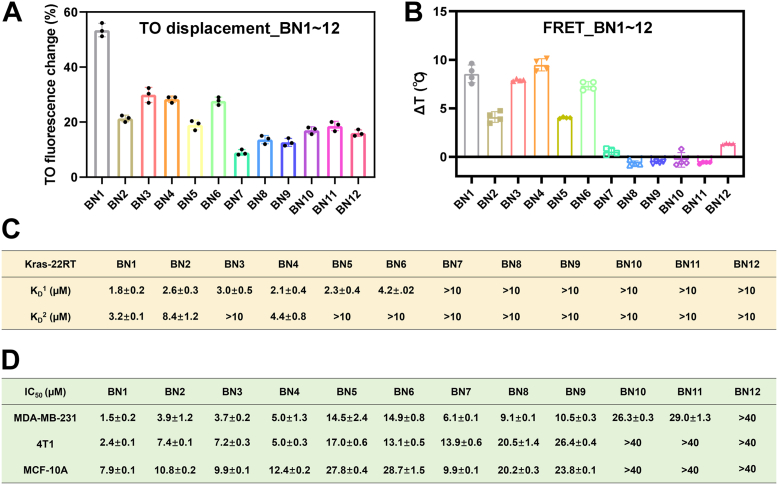
**Screen of indolium-based analogs.***A*, interaction studies of the compounds with KRAS G4 determined by TO displacement assay. *B*, stabilization ability of the compounds on KRAS G4 displayed by Δ*T*_m_ values, determined by FRET assay. *C*, binding affinity between KRAS G4 and the compounds displayed by *K*_D_ values. *K*_D_^1^ and *K*_D_^2^ values were derived from absorption titration and fluorescence quenching assays, respectively. *D*, growth inhibition of the compounds on both tumor cells and normal cells displayed by IC_50_ values, determined by CCK8 assay. The data were presented as mean ± SD (n = 3).

Interaction studies of indolium-based analogs and KRAS G4 were first confirmed by thiazole orange (TO) displacement assay (42). As illustrated, the fluorescence of TO undergoes a substantial increase when it binds to nucleic acids. However, this enhanced fluorescence is subsequently quenched upon the introduction of competitive ligands. Consequently, the degree of TO fluorescence quenching could potentially serve as an indicator of the binding affinities between the tested ligands and nucleic acids. As shown in Figs. 2*A* and S1, TO fluorescence with KRAS G4 was quenched by more than 50% upon the addition of BN1, more dramatically than other indolium-based analogs. FRET assay was further performed to investigate the stabilization ability of the compounds on DNA sequence, where dual-labeled fluorescence KRAS G4 was applied. BN1–BN6 increased *T*_m_ value of KRAS G4, while BN7–BN12 exerted few effects on *T*_m_ value (Fig. 2*B*). BN1 and BN4 were two most effective ligands, inferring that the substitutions of imidazole and thiophene groups enhanced the stability of KRAS G4 with larger aromatic rings. Binding affinity was then presented as *K*_D_^1^ and *K*_D_^2^ values, calculated from absorption titration assay and fluorescence quenching assay, respectively. BN1–BN6 exhibited much stronger binding to KRAS G4 than BN7–BN12, with the most promising ligands as BN1 and BN4, consistent with the FRET results (Figs. 2*C*, S2, and S3).

Cytotoxicity was determined in several types of tumor cells, including MDA-MB-231 cells, 4T1 cells, A549 cells, A549/DDP cells, HepG2 cells, and HCT116 cells. As shown in Figures 2*D* and S4, BN1–BN6 displayed stronger cytotoxicity than BN7–BN12 in tumor cells, and BN1 was the most potent one. Lower cytotoxicity to human normal mammary gland epithelial MCF-10A cells was detected by BN1 than tumor cells, thus BN1 being selected for further studies. Human TNBC MDA-MB-231 cells were the most sensitive cell line to indolium-based analogs with KRAS G13D mutation, while mouse TNBC 4T1 cells exhibited similar behaviors with wild-type KRAS overexpression. Therefore, MDA-MB-231 cells and 4T1 cells were selected for further studies.

### Selectivity of BN1 for KRAS G4

**Figure 3:**
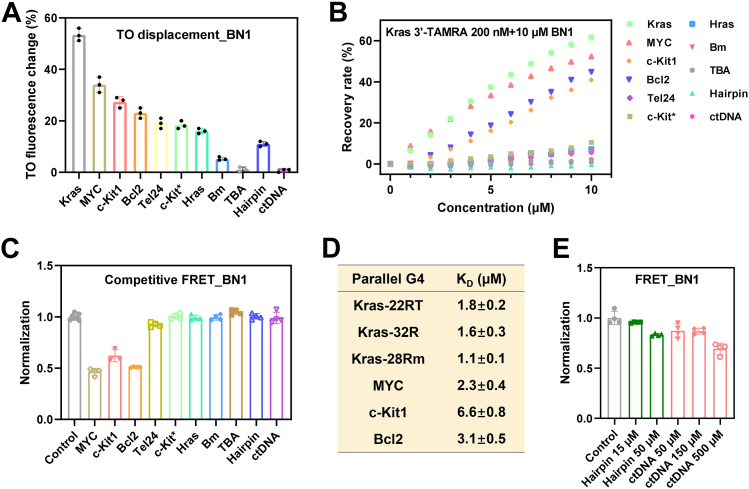
**Selectivity of BN1 for KRAS G4.***A*, interaction studies of BN1 with different types of DNA sequences determined by TO displacement assay. *B*, interaction studies of BN1 with different types of DNA sequences determined by competitive fluorescence recovery assay, where increasing concentrations of unlabeled DNAs were added to 3′-TAMRA-labeled KRAS G4 mixed with BN1. *C*, stabilization ability of BN1 on KRAS G4 determined by competitive FRET assay, where different types of unlabeled DNAs were added to fluorescence-labeled KRAS G4 mixed with BN1. The unlabeled KRAS G4 was used as a positive control. *D*, binding affinity between BN1 and different types of G4 sequences displayed by *K*_D_ values, fitted by Benesi–Hildebrand equation of absorption titration assay. *E*, stabilization ability of BN1 on KRAS G4 determined by competitive FRET assay, where excessive amounts of unlabeled double-stranded DNAs were applied. The unlabeled KRAS G4 was used as a positive control. The data were presented as mean ± SD (n = 3).

The binding selectivity of BN1 to different types of DNAs was first determined by TO displacement assay, including parallel, hybrid, and antiparallel G4s, as well as double-stranded DNAs (Fig. S5). TO fluorescence change was 51.2% of KRAS G4 by BN1, much higher than other parallel G4s (MYC, c-Kit1, Bcl2), non-parallel G4s (Tel 24, c-Kit∗, Hras, Bm, TBA), and double-stranded DNAs (Hairpin, ctDNA) (Fig. 3*A*). Additionally, we conducted a competitive fluorescence recovery assay to investigate the binding selectivity. As shown in Figures 3*B* and S6, the addition of KRAS G4 resulted in the maximum recovery of fluorescence intensity of the solution containing BN1 and 3′-TAMRA-labeled KRAS G4. It was worth noting that only parallel G4s (MYC, c-Kit1, Bcl2) led to significant fluorescence recovery, compared to non-parallel G4s and double-stranded DNAs, further proving the selectivity of BN1 to parallel G4s.

Competitive FRET assay was performed, where other types of DNAs (5 M equivalents) were added to the solution containing BN1 and dual-labeled KRAS G4 (5′-FAM-KRAS-TAMRA-3′, 1 M equivalent). As shown in Figure 3*C*, BN1-increased *T*_m_ value of KRAS G4 remained stable in the presence of non-parallel G4s and double-stranded DNAs, while parallel G4s (MYC, c-Kit1, Bcl2) decreased the stabilization of BN1 on KRAS G4. Similar conclusion was drawn from absorption titration assay, and the results were shown in Figures 3*D* and S7. *K*_D_ values of BN1 with three types of KRAS G4 sequences (Kras-22RT, Kras-32R and Kras-28Rm) were lower than 2 μM, indicating the strong interaction between BN1 and parallel KRAS G4. *K*_D_ values of BN1 with parallel G4s of MYC, c-Kit1 and Bcl2 were 2.3 μM, 6.6 μM, and 3.1 μM, respectively, while *K*_D_ values were too large to be calculated with other types of DNAs. To sum up, BN1 displayed stronger binding selectivity to parallel G4s than non-parallel G4s and double-stranded DNAs. As for parallel G4s, relative binding selectivity of BN1 was demonstrated to KRAS G4 than MYC, c-Kit and Bcl2 G4s.

Finally, we examined the effects of excessive amounts of double-stranded DNAs on BN1-induced stabilization of KRAS G4, where duplex32 was applied at 15, 50 M equivalents of KRAS G4, and calf thymus DNA (ctDNA) was applied at 50, 150, and 500 μM (base-pair concentration) in a competitive FRET assay. Although *T*_m_ value of KRAS G4 reduced to a certain degree with high concentrations of double-stranded DNAs, the stability was not severely disturbed (Fig. 3*E*).

### Binding mode of BN1 and KRAS G4

**Figure 4:**
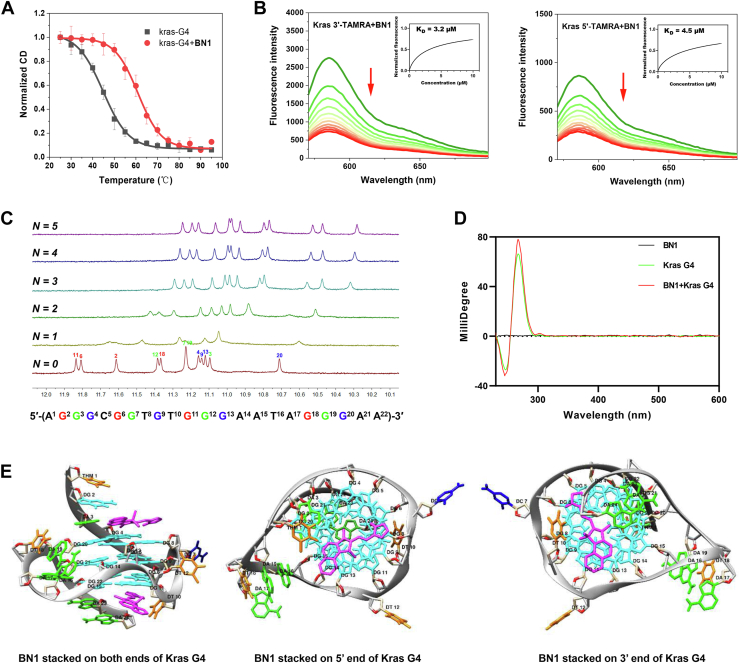
**Binding mode of BN1 and KRAS G4.***A*, stabilization ability of BN1 on KRAS G4 determined by CD melting assay. *B*, fluorescence quenching assay of BN1 with 3′-TAMRA-labeled KRAS G4 or 5′-TAMRA-labeled KRAS G4, where *K*_D_ values were shown in the inset. The fluorescence quenching data were fitted by the Hill equation. *C*, imino region in ^1^H NMR spectra of KRAS G4 in the absence and presence of different molar equivalents of BN1. *D*, CD spectra of KRAS G4 in the absence and presence of BN1. *E*, Molecular docking of the binding between BN1 and KRAS G4 (PDB ID: 7X8M), including side view, *top* view and *bottom* view.

Stabilization of BN1 on KRAS G4 was decided by CD melting assay, where BN1 significantly increased *T*_m_ value of KRAS G4 (Fig. 4*A*), consistent with the results of FRET assay. To fully understand the binding mode of BN1 and KRAS G4, a fluorescence quenching assay was performed with both 3′-TAMRA-labeled and 5′-TAMRA-labeled DNAs. As shown in Figure 4*B*, the fluorescence of KRAS G4 decreased dramatically with the addition of the compound. *K*_D_ values of BN1 with 3′-TAMRA-labeled and 5′-TAMRA-labeled KRAS G4 were 3.2 and 4.5 μM, respectively. The *K*_D_ values at micromolecular level indicated that BN1 could bind to both ends of KRAS G4.

Results of NMR titration assays were shown in Figure 4*C*, where KRAS G4 alone displayed 12 imino proton peaks, in accordance with the presence of three G-tetrads. A batch of imino proton peaks with different chemical shifts were gradually induced after the addition of 1 and 2 M equivalents of BN1. The emerging peaks tended to be saturated and stable after the addition of 3 and more equivalents of BN1, inferring that the binding ratio of BN1 and KRAS G4 could be 2:1. Similar trends were recorded in the aromatic region in ^1^H NMR spectra of KRAS G4 with or without the addition of BN1 (Fig. S8). CD assay results proved that BN1 bound to and maintained the parallel structure of KRAS G4. Moreover, no induced CD signals were recorded, further verifying the end-stacking of BN1 on KRAS G4 (Fig. 4*D*). Finally, the end-stacking mode of BN1 to both 3′- and 5′-ends of KRAS G4 by a ratio of 2:1 was illustrated by molecular docking (Fig. 4*E*) (32).

### KRAS inhibition induced by BN1 in tumor cells

**Figure 5:**
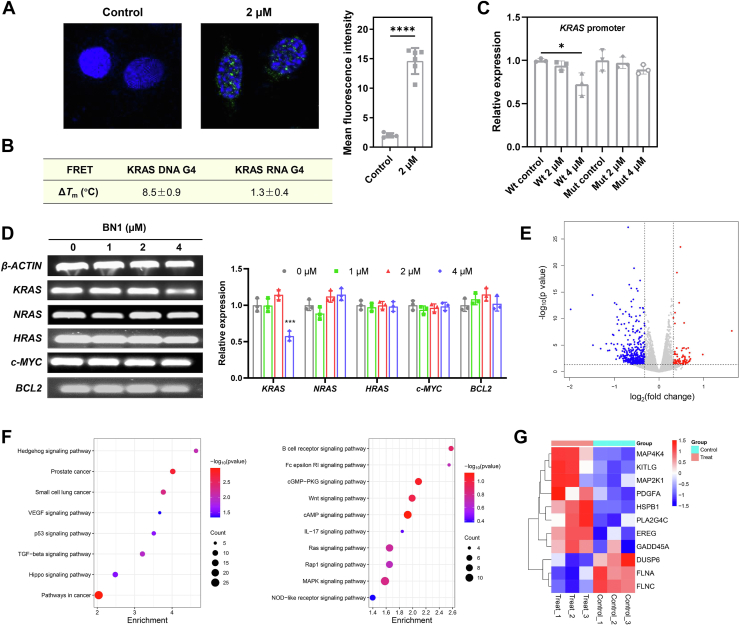
**KRAS inhibition induced by BN1 in MDA-MB-231 cells.***A*, representative immunofluorescence images of BG4 foci (*green*) in BN1-treated cells, with the nuclei stained by DAPI (*blue*). The quantification of BG4 intensity was also displayed. *B*, stabilization ability of BN1 on KRAS RNA G4 determined by FRET melting assay. *C*, relative expression of Renilla luciferase activity to firefly luciferase activity in the plasmids containing wild-type and mutant KRAS promoter in BN1-treated HEK-293T cells. *D*, effects of BN1 on the transcription of G4-related genes in tumor cells determined by RT-PCR assay. *E*, volcano plot of differentially expressed genes in BN1-treated cells, with FDR < 0.05 and fold change > 2 or < 0.5. *F*, KEGG pathway enrichment analysis of differentially expressed genes in BN1-treated cells. *G*, Heatmap plot of differentially expressed genes in MAPK pathway in BN1-treated cells. The data were presented as mean ± SD (n = 3). ∗*p* < 0.05, ∗∗∗*p* < 0.001, ∗∗∗∗*p* < 0.0001 compared to the control group.

Effects of BN1 on KRAS expression were then examined in MDA-MB-231 cells. Representative immunofluorescence images displayed that BN1 triggered remarkable increase of BG4 intensity in the nuclei, proving the G4 formation in the cells (Fig. 5*A*). Notably, a small number of BG4 foci were detected in the extranuclear region. Given that cytoplasmic RNA G4s predominantly adopt parallel conformations, we proposed that these extranuclear BG4 foci may originate from RNA G4 structures. To validate this hypothesis, we performed a FRET melting assay using a fluorophore-labeled KRAS RNA G4 sequence. The results revealed that BN1 modestly enhanced the thermal stability of the RNA G4, although this effect was significantly weaker compared to its stabilization of the KRAS DNA G4 (Fig. 5*B*). Besides, not all green foci in the nuclei were KRAS G4, while other G4s within the genome could be affected by BN1. Dual-luciferase reporter assay was carried out with either wild-type or mutant KRAS promoter, where Renilla luciferase (RL) and Firefly luciferase (FL) are experimental reporter and control reporter, respectively (43). As shown in Figure 5*C*, BN1 decreased the relative expression of RL/FL in HEK-293T cells transfected with wild-type KRAS promoter, but exerted no obvious effects in the cells with mutant KRAS promoter, indicating the importance of G4 structures in the regulation of KRAS expression. Selectivity of BN1 on the KRAS expression was determined by RT-PCR assay, and the results were displayed in Figure 5*D*. BN1 reduced the KRAS mRNA level in MDA-MB-231 cells, while the transcription of other typical G4-related genes remained unchanged, including NRAS, HRAS, c-MYC and BCL2.

RNA sequencing assay was performed to investigate the effects of BN1 on signaling transduction in MDA-MB-231 cells, and reproducibility of the experiment was verified by correlation coefficients and heatmap results (Fig. S9, *A* and *B*). Volcano plot and GO analysis were shown in Figures 5*E* and S9*C*, respectively, where about 600 genes in tumor cells were significantly regulated. KEGG analysis demonstrated BN1 was involved in multiple antitumor pathways, as well as some immune pathways (Fig. 5*F*). Here, MAPK signaling was influenced by BN1, but ranked relatively low among all pathways with significant differences. Nevertheless, MAPK signaling was still discussed by heatmap analysis, since it is the most common KRAS downstream pathway presented in KEGG analysis. Eight genes were upregulated and 3 genes were downregulated in MAPK signaling in BN1-treated MDA-MB-231 cells (Fig. 5*G*).

### Inhibition of KRAS downstream pathways by BN1 in tumor cells

**Figure 6:**
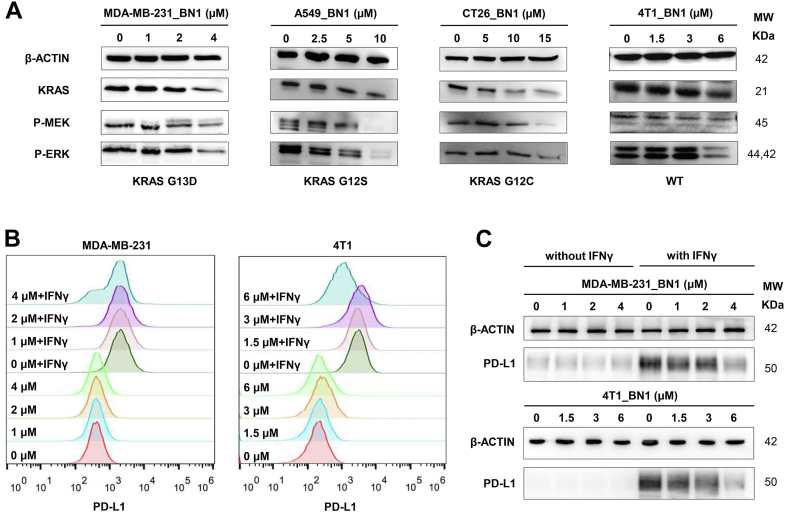
**Inhibition of KRAS downstream pathways by BN1 in tumor cells.***A*, effects of BN1 on MAPK/ERK pathway in different types of tumor cells determined by Western blot assay. *B*, PD-L1 expression in BN1-treated cells stimulated without or with IFNγ determined by flow cytometry. *C*, PD-L1 expression in BN1-treated cells stimulated without or with IFNγ determined by Western blot assay.

The MEK-ERK pathway is a classical KRAS downstream pathway (44, 45), and more than half of the differentially expressed genes in Figure 5*G* were included in this pathway, for example, the MAP2K1 gene (encoding MEK protein). Therefore, the effects of BN1 on the MEK-ERK pathway were fully determined in several types of tumor cells with different KRAS substitutions, namely MDA-MB-231 cells (KRAS G13D mutation), A549 cells (KRAS G12S mutation), CT26 cells (KRAS G12C mutation), and 4T1 cells (KRAS overexpression without mutation). As shown in Figure 6*A*, BN1 dose-dependently suppressed the expression of KRAS, phosphorylated MEK, and phosphorylated ERK in all kinds of tumor cells. Sotorasib, the first drug approved by the FDA targeting the KRAS G12C mutation, was applied as a control agent. As shown in Fig. S10, Sotorasib exhibited potent inhibition on the KRAS-MEK-ERK pathway in CT26 cells but displayed few impacts in other types of tumor cells. In summary, BN1 could possess a wider range of applications than drugs targeting KRAS mutations, since it targeted G4 at the nucleic acid level, rather than at the protein level.

Next, the functions of BN1 on the immune system were investigated, since several related pathways were presented in RNA sequencing results. PD-L1, an immune checkpoint, contributes to the evasion of the host immune system in many cancers, and oncogenic RAS-MEK-ERK signaling is reported to promote PD-L1 expression by stabilizing its mRNA (14). MDA-MB-231 cells and 4T1 cells were treated by BN1 with or without IFNγ stimulation, where IFNγ elevates basal PD-L1 expression in tumor cells (46). As expected, endogenous PD-L1 levels were quite low in two types of tumor cells, while IFNγ remarkably enhanced the expression of PD-L1, as displayed by flow cytometry and Western blot assays. BN1 was relatively inefficient in the regulation of endogenous PD-L1 levels. However, BN1, especially at the highest concentrations (4 μM for MDA-MB-231 cells and 6 μM for 4T1 cells), effectively decreased IFNγ-elevated PD-L1 levels (Fig. 6, *B* and *C*).

### Growth inhibition induced by BN1 in tumor cells

**Figure 7:**
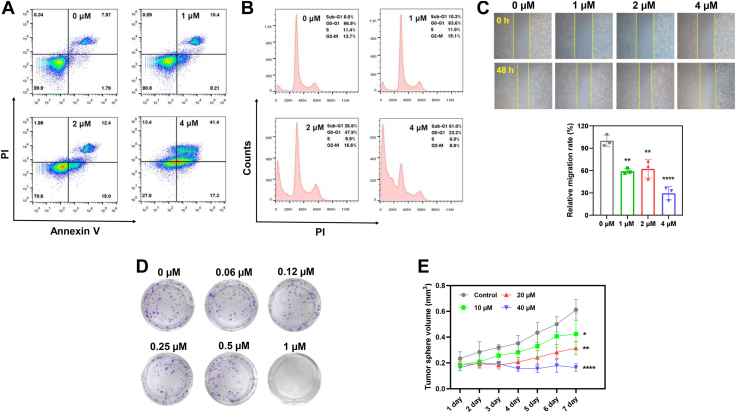
**Growth inhibition induced by BN1 in MDA-MB-231 cells.***A*, effects of BN1 on the induction of apoptosis determined by Annexin V-PI double staining. *B*, effects of BN1 on the induction of cell cycle arrest determined by PI staining. *C*, inhibition of migration in BN1-treated cells determined by wound scrape assay. *D*, growth inhibition in BN1-treated cells determined by colony formation assay. *E*, growth inhibition in BN1-treated cells determined by tumor spheroid assay. The data were presented as mean ± SD (n = 3). ∗*p* < 0.05, ∗∗*p* < 0.01, ∗∗∗∗*p* < 0.0001 compared to the control group.

BN1-induced growth inhibition was first determined in human TNBC MDA-MB-231 cells. BN1 triggered cell apoptosis and cell cycle arrest in G2/M phase in a dose-dependent manner, as examined by flow cytometry (Fig. 7, *A* and *B*). Results of Western blot assay further proved the cleavage of several Caspases and PARP, the increase in BAX (related to apoptosis, Fig. S11*A*), and the decrease in Cyclin B1 (related to G2/M arrest, Fig. S11*B*). Besides, Cyclin D1 (related to G0/G1 arrest) was also inhibited by BN1 to some degree. Antiproliferation and anti-metastasis effects were studied by colony formation and wound scrape assays, respectively. BN1 dramatically decreased the number of visible cell clones (Fig. 7*C*), as well as hindered the repair of the wounded area (Fig. 7*D*). After the assessment of cytotoxicity in 2D monolayer cells, a 3D tumor spheroid assay was performed, which is more similar to the condition of *in vivo* tumor growth. The volumes of tumor spheroids with BN1 treatment for 7 days were significantly smaller than those in the control group (Fig. 7*E*). Inhibition of the viability of tumor spheroids was then verified by Calcein AM/PI staining, where BN1 dose-dependently induced the decrease of green fluorescence (Calcein AM) and the increase of red fluorescence (PI) (Fig. S12).

Moreover, the above effects were explored in mouse TNBC 4T1 cells. BN1 inhibited the expression of KRAS and the following MEK-ERK pathway (Figs 6*A* and S13), leading to cell apoptosis and cell cycle arrest (Fig. S14), with necessary proteins determined by Western blot assay (Fig. S15). Growth inhibition of BN1 on 4T1 cells was then ascertained by colony formation, wound scrape, and tumor spheroid assays (Fig. S16).

### *In vivo* antitumor and immunostimulatory effects of BN1 in tumor-bearing mice

**Figure 8:**
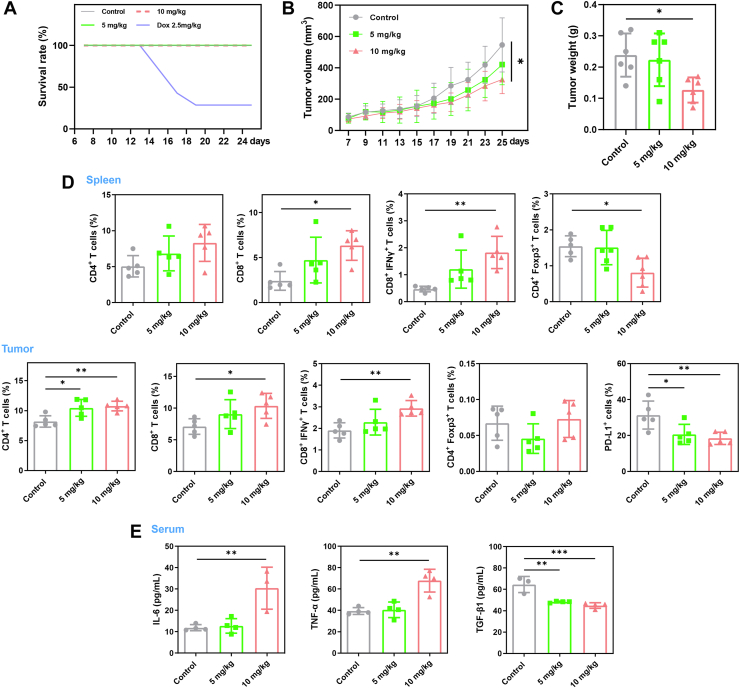
***In vivo* antitumor and immunostimulatory effects of BN1 in 4T1-bearing mice.***A*, survival curves of the mice treated by DOX and BN1. *Grey*, *red* and *green lines* overlapped, since no death was observed in these three groups during the experiment. *B*, growth curves of the tumor volumes measured every other day until Day 25 after tumor implantation (*p* = 0.0126, control vs 10 mg/kg). *C*, tumor weights determined at the time of sacrifice (*p* = 0.0212, control vs 10 mg/kg). *D*, quantitative analyses of CD4^+^, CD4^+^ Foxp3^+^, CD8^+^, CD8^+^ IFNγ^+^ T cells in spleens and tumors, as well as the expression of PD-L1 in tumors, determined by flow cytometry. CD4^+^, CD8^+^ T cells were gated on CD45^+^ CD3^+^ population, and PD-L1 expression was gated on CD45^-^ population. *E*, cytokine levels of IL-6, TNF-α, TGF-β1 in the serum of the mice determined by ELISA. The data were presented as mean ± SD (n ≥ 5). ∗*p* < 0.05, ∗∗*p* < 0.01, ∗∗∗*p* < 0.001 compared to the control group.

*In vivo* antitumor effects were determined in 4T1 tumor-bearing BALB/c mice, where BN1 (5 mg/kg, 10 mg/kg) or DOX (the positive control, 2.5 mg/kg) was given to the mice every other day for 2 weeks. High toxicity of DOX was displayed by the mortality of more than 50% at the end of drug treatment, while all mice in the BN1 group appeared comfortable and healthy (Fig. 8*A*). Therefore, only the results of BN1 were presented afterwards. 10 mg/kg BN1 distinctly inhibited tumor volumes and tumor weights, as shown in Figure 8, *B* and *C*, and S17. Safety of BN1 was further proved, where no significant differences in body weights and organ weights were recorded between the control group and the drug group (Fig. S18, *A* and *B*). In addition, no severe structural and pathological changes of the major organs could be observed in the H&E staining assay (Fig. S18*C*).

Immunostimulatory effects of BN1 were then discussed, since PD-L1 related immune responses were verified *in vitro*. Single cell suspensions of spleens and tumors were prepared, and phenotypes of different cells were investigated by flow cytometry (Fig. S19). As shown in Figures 8*D* and 10 mg/kg BN1 descended PD-L1 expression in tumor cells (CD45^-^ PD-L1^+^), and elevated the percentages of both total CD8^+^ T cells (CD45^+^ CD3^+^ CD8^+^) and activated CD8^+^ T cells (CD45^+^ CD3^+^ CD8^+^ IFNγ^+^) in both spleens and tumors. Total CD4^+^ T cells (CD45^+^ CD3^+^ CD4^+^) were increased, and CD4^+^ Foxp3^+^ T cells (CD45^+^ CD3^+^ CD4^+^ Foxp3^+^) were decreased with BN1 treatment. Thus, BN1 promoted the status of immunotolerance to immunoreaction by the raise of CD8^+^/Foxp3^+^ ratio (47, 48). Immunohistochemistry staining results of Fig. S20 manifested the increase of CD4 and CD8 expression. Besides, KRAS inhibition of BN1 *in vivo* was also detected. Finally, cytokine levels in serum samples were decided by ELISA assay, and the results were shown in Figure 8*E*. BN1 strengthened the levels of typical immunostimulatory cytokines (IL-6 and TNF-α) (49), and weakened the level of typical immunosuppressive cytokine (TGF-β1) (50).

**Figure 9:**
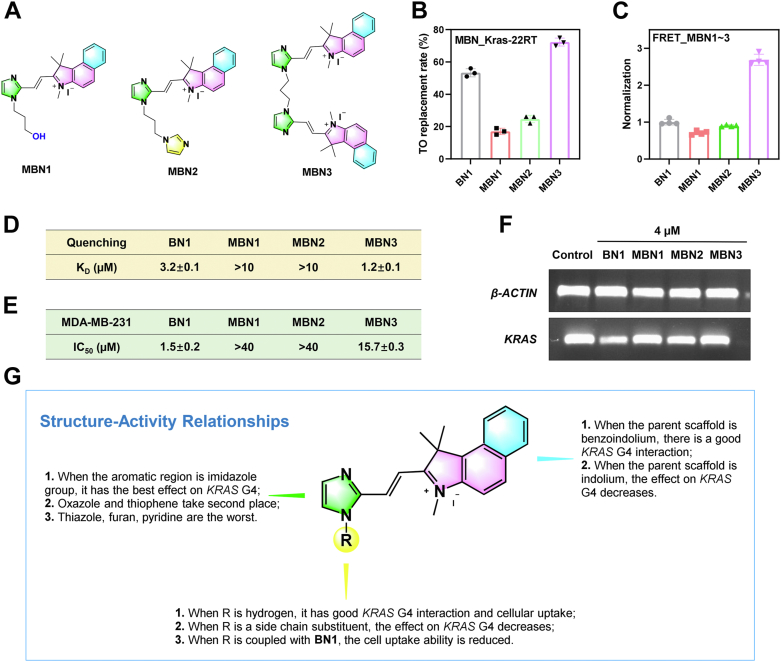
**Modification and bioactivity of BN1 analogs.***A*, chemical structures of BN1 analogs (MBN1–MBN3). *B*, interaction studies of the compounds with KRAS G4 determined by TO displacement assay. *C*, stabilization ability of the compounds on KRAS G4 displayed by Δ*T*_m_ values, determined by FRET assay. *D*, binding affinity between KRAS G4 and the compounds displayed by *K*_D_ values, fitted by nonlinear regression of fluorescence quenching assay. *E*, growth inhibition of the compounds on MDA-MB-231 cells displayed by IC_50_ values, determined by CCK8 assay. *F*, effects of the compounds on the transcription of KRAS in MDA-MB-231 cells determined by RT-PCR assay. The data were presented as mean ± SD (n = 3). *G*, structure–activity relationships of BN1.

**Figure 10:**
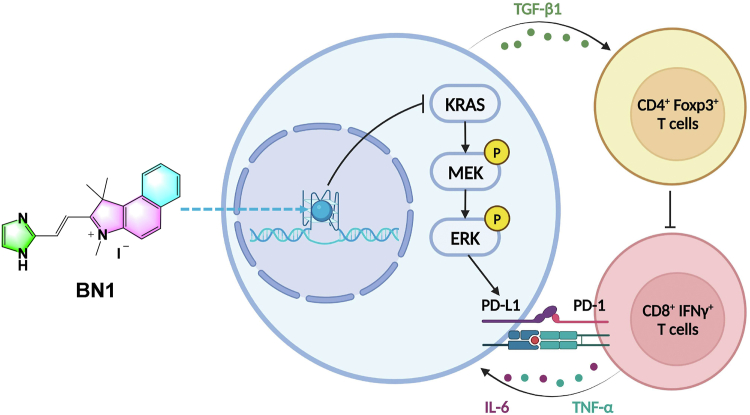
**Schematic illustration of BN1-triggered antitumor immunostimulatory responses**.

### Modification and bioactivity of BN1 analogs

The above experiments demonstrated the promising anticancer activity of BN1 targeting KRAS G4, and thereby, we showed interest in investigating the structure-activity relationships from more dimensions. Thus, we designed and synthesized another three compounds (MBN1–MBN3, Fig. 9*A*) by decorating the NH group on BN1. Then, the binding properties and cytotoxicity were evaluated. As shown in Figs. 9*B* and S21, MBN3 exhibited a higher displacement ratio than BN1 in TO displacement assay, suggesting a more potent binding ability of MBN3 to KRAS G4 than BN1. Stabilization and binding constants were then calculated by FRET and fluorescence quenching assays, respectively. As shown in Figures 9, *C* and *D*, and S22, MBN3 demonstrated much stronger interaction with KRAS G4 than BN1, while MBN1 and MBN2 were weaker than BN1. Nevertheless, the effects of MBN3 on the growth of tumor cells were not satisfactory, where the IC_50_ value was more than 10 μM in MDA-MB-231 cells (Fig. 9*E*), and no obvious reduction of KRAS mRNA level could be detected (Fig. 9*F*). Although the coupling of BN1 enhanced its affinity for KRAS G4, it may be difficult for MBN3 to enter the cells with the increase in molecular dimension and positive charges. Finally, the structure–activity relationships were summarized in Figure 9*G*.

## Conclusion

Targeting G4s in the promoter regions of oncogenes has emerged as an appealing strategy for cancer therapy, while some KRAS G4-directed small molecules have been displayed to suppress gene expression in tumor cells. KRAS mutations and amplifications are commonly present during tumorigenesis, and the canonical downstream pathway is the KRAS-MEK-ERK cascade. Moreover, immunomodulatory effects of KRAS have been unraveled in recent years, where the immune checkpoint, PD-L1, appears as one of its effector proteins. Therefore, more KRAS G4-targeted ligands with novel chemical scaffolds need to be developed, especially with the discussion of pharmacological activities and mechanisms.

We designed and synthesized a small library of BN analogs in this study, and BN1 was chosen as the most promising ligand after the exploration of SAR on KRAS G4. BN1 displayed some selectivity on binding and stabilizing KRAS G4 than other types of DNAs, and the binding ratio was 2:1 by stacking on both ends of KRAS G4. BN1 effectively inhibited KRAS transcription and translation in tumor cells, resulting in the downregulation of phosphorylated MEK and ERK, as well as the induction of cell apoptosis and cell cycle arrest. In addition, suppression on PD-L1 was also detected in BN1-treated MDA-MB-231 cells and 4T1 cells. Antitumor efficacy was then revealed *in vivo*, where BN1 turned to be an effective and safe agent in 4T1-bearing mice. Immunostimulatory mechanisms included the increase of CD8^+^ IFNγ^+^ cells, the decrease of CD4^+^ Foxp3^+^ cells and the regulation of a series of cytokines (Fig. 10). Finally, the structure–activity relationships regarding this scaffold were acquired. In summary, we have developed a drug-like compound BN1 binding toward KRAS G4, which may have good therapeutic potential along with enhanced immunomodulation for wide-spectrum KRAS-driven tumors. However, certain limitations persisted in the current study. The most critical issue lay in the binding selectivity profile of BN1. Although it exhibited enhanced affinity toward parallel G4s compared to non-parallel G4s and double-stranded DNAs, its specificity for the KRAS G4 over other parallel G4s (*e.g.*, MYC, c-Kit1, and Bcl2) remained suboptimal. Therefore, in our future work, we will focus on chemical modification of BN1 or development of other novel molecular scaffolds, aiming to identify ligands with superior KRAS G4-specific binding properties.

## Experimental procedures

### Synthesis and characterization

The 12 designed compounds (BN1–BN12) were easily constructed through coupling the indolium iodide or benzoindolium iodide (1.0 equiv.) and aromatic aldehydes (1.5 equiv.) in EtOH under reflux for 12 h, followed by filtration when cooling without further purification. The 3 analogs of BN1 (MBN1–MBN3) were then prepared through a further substitution reaction, and the details are described below. The structures of all the final compounds were confirmed by ^1^H NMR, ^13^C NMR, MS, and HPLC analysis (>95%) (Figs. S23–S67).

Synthesis of BN1 ((*E*)-2-(2-(1*H*-imidazol-2-yl)vinyl)-1,1,3-trimethyl-1*H*-benzo[*e*]indol-3-ium iodide): yellow solid, 55% yield. ^1^H NMR (600 MHz, CDCl_3_/CD_3_OD) δ 8.45 (d, *J* = 16.4 Hz, 1H), 8.20 (d, *J* = 8.5 Hz, 1H), 8.08 (d, *J* = 8.9 Hz, 1H), 8.03 (d, *J* = 2.6 Hz, 1H), 8.01 (d, *J* = 5.4 Hz, 1H), 7.72 (t, *J* = 7.6 Hz, 1H), 7.67 (d, *J* = 8.9 Hz, 1H), 7.63 (t, *J* = 7.5 Hz, 1H), 7.43 (s, 2H), 4.30 (s, 3H), 2.04 (s, 6H). ^13^C NMR (151 MHz, CDCl_3_/CD_3_OD) δ 182.98, 143.24, 138.90, 138.61, 138.54, 133.83, 131.82, 130.28, 128.85, 127.80, 127.28, 122.85, 111.57, 111.38, 54.06, 35.81, 26.23. MS (ESI) m/z: 302.0 [M-I]^+^.

Synthesis of BN2 ((*E*)-1,1,3-trimethyl-2-(2-(thiazol-2-yl)vinyl)-1*H*-benzo[*e*]indol-3-ium iodide): yellow solid, 50% yield. ^1^H NMR (600 MHz, DMSO) δ 8.72 (d, *J* = 16.1 Hz, 1H), 8.47 (d, *J* = 8.2 Hz, 1H), 8.37–8.28 (m, 3H), 8.25 (d, *J* = 7.9 Hz, 1H), 8.20 (d, *J* = 8.7 Hz, 1H), 7.88 (d, *J* = 16.1 Hz, 1H), 7.84 (t, *J* = 7.3 Hz, 1H), 7.77 (t, *J* = 7.2 Hz, 1H), 4.30 (s, 3H), 2.02 (s, 6H). ^13^C NMR (151 MHz, DMSO) δ 182.35, 162.45, 146.66, 141.01, 139.95, 139.33, 133.92, 131.53, 130.53, 129.04, 128.50, 128.06, 127.07, 123.91, 115.10, 114.02, 54.57, 35.96, 25.13. MS (ESI) m/z: 318.9 [M-I]^+^.

Synthesis of BN3 ((*E*)-1,1,3-trimethyl-2-(2-(oxazol-2-yl)vinyl)-1*H*-benzo[*e*]indol-3-ium iodide): yellow solid, 50% yield. ^1^H NMR (600 MHz, DMSO) δ 8.59 (s, 1H), 8.45 (d, *J* = 8.5 Hz, 1H), 8.35 (d, *J* = 8.9 Hz, 1H), 8.26 (d, *J* = 8.1 Hz, 1H), 8.20 (dd, *J* = 15.0, 12.8 Hz, 2H), 7.84 (ddd, *J* = 8.3, 6.9, 1.3 Hz, 1H), 7.82–7.74 (m, 3H), 4.32 (s, 3H), 1.99 (s, 6H). ^13^C NMR (151 MHz, DMSO) δ 182.37, 159.42, 143.88, 139.92, 139.59, 134.03, 133.10, 131.81, 131.61, 130.53, 129.11, 128.24, 127.02, 123.97, 118.22, 114.11, 54.77, 36.34, 24.68. MS (ESI) m/z: 302.9 [M-I]^+^.

Synthesis of BN4 ((*E*)-1,1,3-trimethyl-2-(2-(thiophen-2-yl)vinyl)-1*H*-benzo[*e*]indol-3-ium iodide): yellow solid, 65% yield. ^1^H NMR (600 MHz, DMSO) δ 8.77 (d, *J* = 16.1 Hz, 1H), 8.43 (d, *J* = 8.3 Hz, 1H), 8.30 (d, *J* = 8.8 Hz, 1H), 8.24–8.18 (m, 3H), 8.11 (d, *J* = 8.8 Hz, 1H), 7.82 (t, *J* = 7.4 Hz, 1H), 7.73 (t, *J* = 7.3 Hz, 1H), 7.43–7.39 (m, 1H), 7.36 (d, *J* = 16.1 Hz, 1H), 4.23 (s, 3H), 2.01 (s, 6H). ^13^C NMR (151 MHz, DMSO) δ 182.30, 145.03, 140.76, 139.91, 138.31, 137.12, 136.71, 133.58, 131.33, 130.51, 130.28, 128.89, 127.57, 127.13, 123.64, 113.72, 111.13, 54.05, 35.25, 25.66. MS (ESI) m/z: 317.9 [M-I]^+^.

Synthesis of BN5 ((*E*)-2-(2-(furan-2-yl)vinyl)-1,1,3-trimethyl-1*H*-benzo[*e*]indol-3-ium iodide): yellow solid, 70% yield. ^1^H NMR (600 MHz, DMSO) δ 8.47–8.39 (m, 2H), 8.33–8.24 (m, 2H), 8.22 (d, *J* = 8.0 Hz, 1H), 8.12 (d, *J* = 8.8 Hz, 1H), 7.81 (t, *J* = 7.4 Hz, 1H), 7.76–7.69 (m, 1H), 7.59 (d, *J* = 2.3 Hz, 1H), 7.30 (d, *J* = 16.2 Hz, 1H), 6.94 (s, 1H), 4.20 (s, 3H), 1.99 (s, 6H). ^13^C NMR (151 MHz, DMSO) δ 182.11, 151.83, 150.49, 139.93, 138.29, 137.24, 133.58, 131.35, 130.50, 128.89, 127.59, 127.15, 123.78, 123.66, 115.36, 113.71, 109.19, 53.91, 35.11, 25.67. MS (ESI) m/z: 302.0 [M-I]^+^.

Synthesis of BN6 ((*E*)-1,1,3-trimethyl-2-(2-(pyridin-2-yl)vinyl)-1*H*-benzo[*e*]indol-3-ium iodide): yellow solid, 60% yield. ^1^H NMR (600 MHz, DMSO) δ 8.85 (d, *J* = 4.6 Hz, 1H), 8.53 (d, *J* = 16.1 Hz, 1H), 8.48 (d, *J* = 8.5 Hz, 1H), 8.34 (d, *J* = 8.9 Hz, 1H), 8.26 (d, *J* = 8.1 Hz, 1H), 8.21 (d, *J* = 8.9 Hz, 1H), 8.19 (d, *J* = 7.7 Hz, 1H), 8.11–8.05 (m, 2H), 7.84 (ddd, *J* = 8.3, 6.9, 1.2 Hz, 1H), 7.80–7.75 (m, 1H), 7.62 (ddd, *J* = 7.6, 4.6, 1.1 Hz, 1H), 4.31 (s, 3H), 2.04 (s, 6H). ^13^C NMR (151 MHz, DMSO) δ 182.96, 152.02, 151.07, 149.62, 139.92, 139.20, 138.25, 133.92, 131.50, 130.53, 129.03, 128.48, 128.02, 127.09, 127.00, 123.89, 115.65, 114.05, 54.65, 35.95, 25.24. MS (ESI) m/z: 313.0 [M-I]^+^.

Synthesis of BN7 ((*E*)-2-(2-(1H-imidazol-2-yl)vinyl)-1,3,3-trimethyl-3*H*-indol-1-ium iodide): yellow solid, 50% yield. ^1^H NMR (600 MHz, DMSO) δ 8.16 (d, *J* = 16.2 Hz, 1H), 7.92 (d, *J* = 8.1 Hz, 1H), 7.87 (d, *J* = 6.7 Hz, 1H), 7.71–7.54 (m, 5H), 4.07 (s, 3H), 1.76 (s, 6H). ^13^C NMR (151 MHz, DMSO) δ 181.55, 143.90, 143.75, 142.34, 139.29, 129.73, 129.50, 123.38, 115.53, 111.60, 52.33, 34.79, 25.99. MS (ESI) m/z: 252.0 [M-I]^+^.

Synthesis of BN8 ((*E*)-1,3,3-trimethyl-2-(2-(thiazol-2-yl)vinyl)-3*H*-indol-1-ium iodide): yellow solid, 60% yield. ^1^H NMR (600 MHz, DMSO) δ 8.64 (d, *J* = 16.1 Hz, 1H), 8.30 (dd, *J* = 7.0, 3.0 Hz, 2H), 8.03–8.00 (m, 1H), 7.94–7.90 (m, 1H), 7.82 (d, *J* = 16.0 Hz, 1H), 7.70–7.65 (m, 2H), 4.17 (s, 3H), 1.80 (s, 6H). ^13^C NMR (151 MHz, DMSO) δ 181.80, 162.28, 146.75, 144.38, 142.30, 141.89, 130.52, 129.60, 128.79, 123.49, 116.19, 115.48, 52.99, 35.42, 25.27. MS (ESI) m/z: 269.0 [M-I]^+^.

Synthesis of BN9 ((*E*)-1,3,3-trimethyl-2-(2-(oxazol-2-yl)vinyl)-3*H*-indol-1-ium iodide): yellow solid, 75% yield. ^1^H NMR (600 MHz, DMSO) δ 8.58 (s, 1H), 8.14 (d, *J* = 16.5 Hz, 1H), 8.07–8.02 (m, 1H), 7.96–7.90 (m, 1H), 7.76–7.65 (m, 4H), 4.20 (s, 3H), 1.77 (s, 6H). ^13^C NMR (151 MHz, DMSO) δ 181.78, 159.36, 144.48, 144.01, 142.25, 133.84, 131.89, 130.85, 129.68, 123.56, 118.67, 116.51, 53.25, 35.82, 24.91. MS (ESI) m/z: 253.0 [M-I]^+^.

Synthesis of BN10 ((*E*)-1,3,3-trimethyl-2-(2-(thiophen-2-yl)vinyl)-3*H*-indol-1-ium iodide): yellow solid, 75% yield. ^1^H NMR (600 MHz, DMSO) δ 8.69 (d, *J* = 16.0 Hz, 1H), 8.23–8.18 (m, 2H), 7.91–7.86 (m, 2H), 7.62 (pd, *J* = 7.5, 1.3 Hz, 2H), 7.40 (dd, *J* = 4.8, 3.9 Hz, 1H), 7.31 (d, *J* = 16.0 Hz, 1H), 4.10 (s, 3H), 1.78 (s, 6H). ^13^C NMR (151 MHz, DMSO) δ 181.62, 146.06, 143.88, 142.30, 140.70, 137.55, 137.12, 130.31, 129.60, 129.42, 123.33, 115.44, 111.52, 52.43, 34.76, 25.85. MS (ESI) m/z: 268.0 [M-I]^+^.

Synthesis of BN11 ((*E*)-2-(2-(furan-2-yl)vinyl)-1,3,3-trimethyl-3*H*-indol-1-ium iodide): yellow solid, 60% yield. ^1^H NMR (600 MHz, DMSO) δ 8.35 (d, *J* = 16.1 Hz, 1H), 8.26 (d, *J* = 1.4 Hz, 1H), 7.93–7.89 (m, 1H), 7.88–7.85 (m, 1H), 7.62 (pd, *J* = 7.5, 1.3 Hz, 2H), 7.58 (d, *J* = 3.5 Hz, 1H), 7.24 (d, *J* = 16.1 Hz, 1H), 6.93 (dd, *J* = 3.6, 1.7 Hz, 1H), 4.08 (s, 3H), 1.76 (s, 6H). ^13^C NMR (151 MHz, DMSO) δ 181.44, 151.81, 150.73, 143.81, 142.31, 138.18, 129.64, 129.44, 124.24, 123.35, 115.45, 115.41, 109.55, 52.29, 34.62, 25.82. MS (ESI) m/z: 252.0 [M-I]^+^.

Synthesis of BN12 ((*E*)-1,3,3-trimethyl-2-(2-(pyridin-2-yl)vinyl)-3*H*-indol-1-ium iodide): yellow solid, 50% yield. ^1^H NMR (600 MHz, DMSO) δ 8.84 (d, *J* = 4.5 Hz, 1H), 8.47 (d, *J* = 16.0 Hz, 1H), 8.16 (d, *J* = 7.7 Hz, 1H), 8.06 (td, *J* = 7.7, 1.7 Hz, 1H), 8.04–7.99 (m, 2H), 7.96–7.91 (m, 1H), 7.70–7.65 (m, 2H), 7.63–7.56 (m, 1H), 4.18 (s, 3H), 1.82 (s, 6H). ^13^C NMR (151 MHz, DMSO) δ 182.36, 151.90, 151.11, 150.60, 144.31, 142.29, 138.25, 130.47, 129.58, 128.70, 127.13, 123.52, 116.21, 116.04, 53.08, 35.41, 25.43. MS (ESI) m/z: 263.0 [M-I]^+^.

Synthesis of MBN1 ((*E*)-2-(2-(1-(3-hydroxypropyl)-1*H*-imidazol-2-yl)vinyl)-1,1,3-trimethyl-1*H*-benzo[*e*]indol-3-ium iodide): BN1 (1.0 mmol), 3-iodopropan-1-ol (1.2 mmol), and K_2_CO_3_ (0.5 mmol) were added to 20 ml of acetonitrile, and the mixture was stirred at 80 °C for 24 h. Afterwards, the solvent was removed and the crude product was purified by column chromatography (CH_2_Cl_2_:MeOH = 20:1). Then, the final product was obtained as a yellow solid, 45% yield. ^1^H NMR (600 MHz, DMSO) δ 8.41 (d, *J* = 8.5 Hz, 1H), 8.31 (d, *J* = 8.9 Hz, 1H), 8.23 (d, *J* = 8.2 Hz, 1H), 8.17 (d, *J* = 3.3 Hz, 1H), 8.15 (d, *J* = 3.4 Hz, 1H), 7.82 (t, *J* = 7.1 Hz, 1H), 7.77 (s, 1H), 7.76–7.69 (m, 2H), 7.47 (s, 1H), 4.86 (s, 1H), 4.48 (t, *J* = 6.7 Hz, 2H), 4.22 (s, 3H), 3.40–3.36 (m, 2H), 2.01 (s, 6H), 1.93 (p, *J* = 6.3 Hz, 2H). ^13^C NMR (151 MHz, DMSO) δ 182.09, 143.27, 139.98, 138.47, 134.93, 133.67, 133.65, 131.38, 130.52, 128.95, 127.73, 127.67, 127.15, 123.61, 113.83, 111.98, 57.17, 54.05, 43.03, 35.34, 34.41, 25.70. MS (ESI) m/z: 359.9 [M-I]^+^.

Synthesis of MBN2 ((*E*)-2-(2-(1-(3-(1*H*-imidazol-1-yl)propyl)-1*H*-imidazol-2-yl)vinyl)-1,1,3-trimethyl-1*H*-benzo[*e*]indol-3-ium iodide): BN1 (1.0 mmol), 1-(3-iodopropyl)-1*H*-imidazole (1.2 mmol), and K_2_CO_3_ (0.5 mmol) were added to 20 ml of acetonitrile, and the mixture was stirred at 80 °C for 24 h. Afterwards, the solvent was removed and the crude product was purified by column chromatography (CH_2_Cl_2_:MeOH = 20:1). Then, the final product was obtained as a yellow solid, 35% yield. ^1^H NMR (600 MHz, DMSO) δ 8.40 (d, *J* = 8.5 Hz, 1H), 8.31 (d, *J* = 8.9 Hz, 1H), 8.24 (d, *J* = 8.2 Hz, 1H), 8.16 (d, *J* = 8.9 Hz, 1H), 7.94 (d, *J* = 15.6 Hz, 1H), 7.83 (t, *J* = 8.2 Hz, 1H), 7.78 (d, *J* = 5.9 Hz, 2H), 7.74 (t, *J* = 7.2 Hz, 1H), 7.70 (d, *J* = 15.6 Hz, 1H), 7.47 (s, 1H), 7.29 (s, 1H), 7.01 (s, 1H), 4.41 (t, *J* = 7.3 Hz, 2H), 4.22 (s, 3H), 4.09 (t, *J* = 7.1 Hz, 2H), 2.36–2.26 (m, 2H), 1.97 (s, 6H). ^13^C NMR (151 MHz, DMSO) δ 182.09, 142.92, 140.02, 138.41, 137.69, 134.28, 133.68, 133.64, 131.41, 130.53, 129.00, 128.77, 127.73, 127.43, 127.16, 123.61, 119.85, 113.86, 112.39, 54.11, 43.94, 43.73, 35.56, 32.69, 25.62. MS (ESI) m/z: 410.0 [M-I]^+^.

Synthesis of MBN3 (2,2'-((1*E*,1′*E*)-(propane-1,3-diylbis(1*H*-imidazole-1,2-diyl))bis(ethene-2,1-diyl))bis(1,1,3-trimethyl-1*H*-benzo[*e*]indol-3-ium) iodide): BN1 (1.0 mmol), 1,3-diiodopropane (0.6 mmol) and K_2_CO_3_ (0.5 mmol) were added into 20 ml of acetonitrile, and the mixture was stirred at 80 °C for 24 h. Afterwards, the solvent was removed and the crude product was purified by column chromatography (CH_2_Cl_2_:MeOH = 20:1). Then, the final product was obtained as yellow solid, 50% yield. ^1^H NMR (600 MHz, DMSO) δ 8.41 (d, *J* = 8.5 Hz, 2H), 8.32 (d, *J* = 8.9 Hz, 2H), 8.24 (d, *J* = 8.2 Hz, 2H), 8.17 (d, *J* = 8.9 Hz, 2H), 8.01 (d, *J* = 15.6 Hz, 2H), 7.88–7.80 (m, 4H), 7.78–7.71 (m, 4H), 7.54 (s, 2H), 4.54 (t, *J* = 7.2 Hz, 4H), 4.23 (s, 6H), 2.44–2.36 (m, 2H), 2.00 (s, 12H). ^13^C NMR (151 MHz, DMSO) δ 182.03, 143.02, 140.05, 138.40, 134.29, 133.80, 133.70, 131.44, 130.56, 129.00, 127.76, 127.28, 127.15, 123.60, 113.89, 112.47, 54.14, 43.72, 35.63, 33.02, 25.70. MS (ESI) m/z: 322.0 [M-2I]^2+^.

### Materials

Compounds were dissolved in DMSO at 10 mM for stock solutions and diluted in related buffers for different experiments. Equal molar concentrations of DMSO were diluted in buffers for the control group. All DNAs were dissolved in Tris-HCl buffer, and their concentrations were determined based on absorbance at 260 nm using a NanoDrop 1000 spectrophotometer (Thermo Scientific). The base-pair concentration of the stock ctDNA solution was determined spectrophotometrically at 260 nm by using the molar extinction coefficient of 13,200 M^−1^ cm^−1^ (per base pair). To obtain G4 structures, DNAs were annealed in relevant buffers containing 100 mM KCl by heating at 95 °C for 5 min, followed by gradual cooling to room temperature. G4 formation was determined by a circular dichroism (CD) spectrophotometer.

### TO displacement assay

TO displacement assay was performed on a Hitachi F-4600 fluorescence spectrophotometer, where the intensity of the mixture of TO probe (0.5 μM) and DNA sequences (0.25 μM) in Tris-HCl buffer (10 mM, pH 7.4) with KCl (100 mM) was defined as F_0_. Then, the mixture was added with small amounts of a stock solution of the compounds at a final concentration of 1 μM, and fluorescence spectra were recorded after each addition. F_10_ was the fluorescence intensity of the solution with 10 μM compounds (after 10 times addition), and the TO fluorescence change was calculated as (F_0_-F_10_)/F_0_ × 100%. Three measurements were conducted in the assay.

### FRET assay

FRET assay was performed on a real-time PCR apparatus (Roche, Switzerland), where dual-labeled fluorescence KRAS DNA sequence (5′-FAM-KRAS-TAMRA-3′, 1 μM) was incubated with the compounds (10 μM) in Tris-HCl buffer (10 mM, pH 7.4) with KCl (10 mM) at room temperature for 2 h. For FRET competitive assay, other types of unlabeled DNA sequences were further added at 5 μM, while hairpin and ctDNA were added at even higher concentrations of 15 μM, 50 μM, 150 μM and 500 μM. The fluorescence intensity of FAM was recorded in the range of 37 °C to 95 °C (excitation wavelength at 470 nm, emission wavelength at 514 nm), and *T*_m_ values were acquired by fitting the data with Boltzmann equation. Four measurements were conducted in the assay.

### Absorption titration assay

Absorption titration assay was performed on an Agilent Cary 60 spectrophotometer, where the compounds (5 μM) were added with small amounts of a stock solution of DNA sequences at a final concentration of 1 μM in Tris-HCl buffer (10 mM, pH 7.4) with KCl (100 mM). Three measurements were conducted in the assay. The absorbance spectra were recorded after each addition, and *K*_D_ values were fitted with the Benesi-Hildebrand equation (51):

$$\frac{1}{A-A_{0}}=\frac{1}{K_{a}\left(A_{\max}-A_{0}\right)\left[DNA\right]}+\frac{1}{A_{\max}-A_{0}}$$

Where *A* is the experimentally measured absorption intensity, *A*_0_ is the absorption intensity of free BN-1, and *A*_max_ is the saturated absorption intensity of the BN-1/DNA complex. The association constant (*K*_a_) was evaluated by plotting 1/[*A*–*A*_0_] *versus* 1/[DNA].

### Fluorescence quenching assay

Fluorescence quenching assay was performed on a Hitachi F-4600 fluorescence spectrophotometer, where 3′-TAMRA-labeled and sometimes 5′-TAMRA-labeled KRAS DNA sequences were applied. KRAS DNA sequence (200 nM) was added with small amounts of a stock solution of the compounds at a final concentration of 1 μM in Tris-HCl buffer (10 mM, pH 7.4) with KCl (100 mM), and fluorescence spectra were recorded after each addition. Three measurements were conducted in the assay. *K*_D_ values were fitted with the Hill equation. For a competitive fluorescence recovery assay, other types of unlabeled DNA sequences were further added to the above solution (200 nM fluorescence KRAS sequence with 10 μM compound), until the final concentration of unlabeled DNA sequences reached 10 μM. Recovery rate was calculated as (F_10’_-F_10_)/(F_0_-F_10_) × 100%, where F_0_ was the fluorescence intensity of KRAS alone. F_10_ was the fluorescence intensity of KRAS with 10 μM compound, and F_10’_ was the fluorescence intensity of KRAS with 10 μM compound and 10 μM other DNAs.

### CD assay

CD assay was performed on a Chirascan CD spectrophotometer, where the solution of 2 μM KRAS DNA sequence with or without 10 μM BN1 in Tris-HCl buffer (10 mM, pH 7.4) with KCl (10 mM) was recorded. For CD melting assay, the solution of 2 μM KRAS DNA sequence with or without 6 μM BN1 was recorded from 25 °C to 95 °C. Three measurements were conducted in the assay.

### NMR titration assay

NMR titration assay was performed on a Bruker 600 MHz NMR spectrometer, where the solution of 200 μM KRAS G4 DNA sequence containing 10% D_2_O in KH_2_PO_4_/K_2_HPO_4_ buffer (25 mM, pH 7.4) with KCl (70 mM) was recorded, added with different amounts of BN1 (from 1:1 to 1:5).

### Cytotoxicity assay

Different types of cells were cultured in 96-well plates (5000 cells/well) and treated with the compounds for 24 h. Cells were incubated with CCK8 solution for 2 h, and OD values at 450 nm were measured with the spectrophotometer (BioTek). IC_50_ values were determined by plotting cell viability *versus* drug dose.

### Docking studies

Molecular docking was performed using the Glide program from Schrödinger Inc. The structure of Kras G4 was retrieved from the PDB database (PDB ID: 7X8M), and the binding sites were defined based on this structure. The low-energy 3D structure of the ligand was generated using LigPrep within Maestro. The protonation state of the ligand was determined at a pH of 7.0. Subsequently, the ligand was docked into the Kras G4 structure using the Ligand Docking panel in Maestro.

### Immunofluorescence assay

MDA-MB-231 cells were cultured in glass-bottom cell culture dishes (1.5 × 10^5^ cells/dish) and treated with 2 μM BN1 for 24 h. At the end of treatment, the cells were fixed with 4% paraformaldehyde for 15 min and permeabilized with 0.3% Triton X-100 for 30 min. Immunofluorescence was performed by incubating the cells with BG4, anti-FLAG antibody (Abcam) at 4 °C overnight, and then with Alexa Fluor 488-labeled anti-rabbit IgG antibody (Abcam) at 37 °C for 3 h. Finally, the cells were incubated with DAPI for 10 min in the dark, and the fluorescence images were captured by a confocal laser scanning microscope (ZEISS LSM 900).

### Dual-luciferase reporter assay

HEK-293T cells were cultured in 96-well plates (10,000 cells/well), and transfected with the plasmids of psiCHECK-2 vectors (Promega) inserted with a wild-type or mutant KRAS promoter for 24 h, and treated with BN1 for another 24 h. Luciferase activity was measured with the Dual-Luciferase Reporter Assay System (Promega), according to the supplier’s instructions, on a Synergy H1 multi-mode microplate reader (BioTek). Renilla luciferase activity was normalized by Firefly luciferase activity.

### RT-PCR assay

MDA-MB-231 cells or 4T1 cells were cultured in 6-well plates (3 × 10^5^ cells/well) and treated with the compounds for 24 h. Total RNA was extracted with Cell Total RNA Isolation Kit (Foregene, China), according to the supplier’s instruction. Reverse transcription was completed with Transcriptor First Strand cDNA Synthesis Kit (Roche, Switzerland), according to the supplier’s instruction, to harvest cDNA. Afterwards, PCR was performed on a PCR device (Thermo Scientific), and the program for all genes included a denaturing cycle (95 °C for 5 min) and 28 PCR cycles (95 °C for 30 s, 58 °C for 30 s and 72 °C for 40 s). Sequences of the PCR primers were listed in Table S2.

### Western blot assay

MDA-MB-231 cells or 4T1 cells were cultured in 6-well plates (3 × 10^5^ cells/well) and treated with BN1 for 24 h. Total protein was extracted with RIPA buffer containing protease inhibitors and phosphatase inhibitors. 20 to 30 μg protein was separated by SDS-PAGE and transferred to a PVDF membrane. The membrane was incubated with anti-human/mouse KRAS, P-MEK, P-ERK, Cleaved Caspase 3, Cleaved Caspase 7, Cleaved Caspase 9, PARP, BAX, Cyclin B1, Cyclin D1, PD-L1, and β-actin antibodies (CST) overnight at 4 °C and the peroxidase-conjugated secondary antibodies (CST) for 1 h at room temperature. Protein bands were detected by the Enhanced Chemiluminescence Kit (Bio-Rad) and captured by an imaging system (CLiNX).

### RNA sequencing assay

MDA-MB-231 cells were cultured in 6-well plates (3 × 10^5^ cells/well) and treated with BN1 for 24 h, while cells in the control group were treated with culture medium supplemented with an equal molar concentration of DMSO as the drug treatment group. Total RNA was extracted using TRIzol Reagent (Invitrogen). 2 μg total RNA was used for stranded RNA sequencing library preparation using KC Stranded mRNA Library Prep Kit for Illumina (Wuhan Seqhealth, China), according to the supplier’s manual. PCR products corresponding to 200 to 500 bp were enriched, quantified, and sequenced on the DNBSEQ-T7 sequencer (MGI Tech). Raw sequencing data were filtered, and clean data were mapped to the reference genome of *Homo sapiens* using STRA software. The total number of transcripts identified in this analysis was about 1.6 × 10^5^. *p*-value cutoff of 0.05 and fold-change cutoff of 2 were used to judge the statistical significance of gene expression differences. As for the differentially expressed genes, 97 genes were upregulated and 465 genes were downregulated with the treatment of BN1. GO enrichment and KEGG pathway enrichment analyses were performed using the Database for Annotation, Visualization and Integrated Discovery (DAVID) (https://david.ncifcrf.gov/). KEGG analysis was based on upregulated and downregulated differentially expressed genes. Data analysis and visualization were performed on the online platform (https://www.bioinformatics.com.cn) (Shanghai NewCore Biotechnology, China). Raw sequencing data were deposited in GEO with the accession number GSE280939.

### Flow cytometry assay

MDA-MB-231 cells or 4T1 cells were cultured in 6-well plates (3 × 10^5^ cells/well) and treated with BN1 for 24 h. For the detection of PD-L1, cells were collected and stained with anti-PD-L1-APC (BioLegend) for 20 min at 4 °C. Apoptosis and cell cycle analyses were determined by Annexin V/PI Apoptosis Detection Kit and Cell Cycle Detection Kit (Dojindo), respectively, according to the supplier’s manual. The fluorescence was quantitated by the flow cytometry (Thermo Fisher Scientific).

### Wound scrape assay

MDA-MB-231 cells or 4T1 cells were cultured in 6-well plates (5 × 10^5^ cells/well) until a confluent single layer of the cells was formed. A vertical line on the bottom of the plate was produced to form a small wound area. BN1 was diluted in serum-free medium and added for 48 h. Images were captured after rinsing the plate with PBS. Migration rate = (0 h scratch width - 48 h scratch width)/0 h scratch width × 100%, and relative migration rates were normalized to the control group (100%).

### Colony formation assay

MDA-MB-231 cells or 4T1 cells were cultured in 6-well plates (800 cells/well) and treated with BN1 for 7 days. Cells were fixed with 4% paraformaldehyde and stained with crystal violet. Images were captured after rinsing the plate with PBS.

### Tumor spheroid assay

MDA-MB-231 cells or 4T1 cells were cultured in 96-well round-bottom ultra-low attachment plates (2500 cells/well) until tumor spheroids were formed, and then treated with BN1 for 7 days. Viability of tumor spheroids was determined by Calcein AM/PI Double Staining Kit (Dojindo), according to the supplier’s instructions.

### *In vivo* tumor implantation study

On Day 0, 4T1 cells (2 × 10^5^ cells/mice) were subcutaneously inoculated into the right flanks of female 6-week-old BALB/c mice. On Day 7, the mice were randomly divided into the Control group, BN1 group (5 mg/kg), and BN2 group (10 mg/kg), and administered with drugs every other day. Tumor dimensions and body weights were measured every other day, and tumor volumes were calculated as (length × width × width)/2. On Day 21, the tumors were removed, photographed, and weighed. Hearts, livers, lungs, and kidneys were harvested and weighed, while H&E staining of the above major organs was performed. Single cell suspensions of the spleens and the tumors were prepared, where the tumors were additionally incubated with collagenase D and DNase I (Sigma-Aldrich) at 37 °C for 1 h. To identify the subsets, cells were first stained with anti-CD45-BV605, anti-CD3-FITC, anti-CD8a-PerCP/Cy5.5, anti-CD4-PE, and anti-PD-L1-APC (BioLegend) for surface staining and then stained with anti-IFNγ-BV421 and anti-Foxp3-Alexa Fluor 700 (BioLegend) for intracellular staining and nuclear staining, respectively. All the above cell samples were determined by flow cytometry (Thermo Fisher Scientific). Immunohistochemistry staining was also used to analyze CD8, CD4, and KRAS. An ELISA assay was used to analyze the cytokine levels in serum samples. The animal experiments were approved by the Animal Ethics Committee of Shenzhen University.

### Statistical analysis

The data were plotted and expressed as the mean ± SD for three independently performed experiments. The data were statistically analyzed using one-way ANOVA with Tukey’s *post hoc* test, and the value of *p* < 0.05 was statistically significant.

## Data availability

Data are available from the authors upon request.

## Supporting information

This article contains supporting information.

## Conflict of interest

The authors declare that they have no known competing financial interests or personal relationships that could have appeared to influence the work reported in this paper.
