# Supplementary material for: A small-sized imidazole-derived ligand binds to the KRAS promoter G-quadruplex and inhibits cancer growth with enhanced immunomodulation
Source: J Biol Chem. 2025 Aug 28;301(10):110647. doi: 10.1016/j.jbc.2025.110647 (PMC12494555; doi:10.1016/j.jbc.2025.110647)
Supplement: Supplementary Data [file mmc1.docx]

**A small-size imidazole-derived ligand binds to the KRAS promoter G-quadruplex and inhibits cancer growth with enhanced immunomodulation**

Xiao-Dong Wang^†^, Qian-Wen Nie^†^, Ming-Hao Hu*

Nation-Regional Engineering Lab for Synthetic Biology of Medicine, International Cancer Center, School of Pharmacy, Shenzhen University Medical School, Shenzhen 518060, China

^†^ These authors contributed equally.

* Corresponding author, humhao1229@szu.edu.cn

**Table of Contents:**

| **Table S1.** Oligonucleotides used in this study | S3 |
| --- | --- |
| **Table S2.** Primers used in this study | S3 |
| **Figure S1.** TO displacement assay of indolium-based analogs and KRAS G4 | S4 |
| **Figure S2.** Absorption titration assay of indolium-based analogs and KRAS G4 | S5 |
| **Figure S3.** Fluorescence quenching assay of ligands and 3’-TAMRA-labeled KRAS G4 | S6 |
| **Figure S4.** Growth inhibition of indolium-based analogs on different types of tumor cells | S6 |
| **Figure S5.** Normalized CD spectra of the tested G4s in this study | S7 |
| **Figure S6.** Competitive fluorescence recovery assay performed by increasing concentrations of different types of unlabeled DNAs added to 3’-TAMRA-labeled KRAS G4 mixed with **BN1** | S8 |
| **Figure S7.** Absorption titration assay of **BN1** and different types of DNAs | S9 |
| **Figure S8.** Aromatic region in ^1^H NMR spectra of KRAS G4 in the absence and presence of different molar equivalents of **BN1** | S10 |
| **Figure S9.** Additional data from RNA seq, including heatmap diagram, cluster analysis and GO categorization | S10 |
| **Figure S10.** Effects of Sotorasib on MAPK/ERK pathway in different types of tumor cells | S11 |
| **Figure S11.** Effects of **BN1** on apoptosis, cell cycle-related proteins in MDA-MB-231 cells | S11 |
| **Figure S12.** Images of MDA-MB-231 tumor spheroids stained with Calcein AM and PI | S11 |
| **Figure S13.** Effects of **BN1** on transcription of KRAS, expression of MEK and ERK in 4T1 cells | S12 |
| **Figure S14.** Effects of **BN1** on the induction of apoptosis and cell cycle arrest in 4T1 cells | S12 |
| **Figure S15.** Effects of **BN1** on apoptosis, cell cycle-related proteins in 4T1 cells | S13 |
| **Figure S16.** Inhibition of 4T1 cell migration, growth by **BN1** determined by wound scrape assay, colony formation assay, tumor spheroid assay | S13 |
| **Figure S17.** Images of the excised tumors from all the groups in tumor implantation | S13 |
| **Figure S18.** Body weights of the mice measured every other day, weights of the major organs of the mice, H&E staining images of the major organs of the mice | S14 |
| **Figure S19.** Flow cytometric plots of CD4^+^, CD8^+^, CD4^+^ Foxp3^+^, CD8^+^ IFNγ^+^ T cells and PD-L1^+^ cells, from both control group and **BN1** group | S15 |
| **Figure S20.** Representative immunohistochemistry staining images for CD4, CD8, KRAS | S15 |
| **Figure S21.** TO displacement assay of **MBN1** to **MBN3** and KRAS G4 | S16 |
| **Figure S22.** Fluorescence quenching assay of **MBN1** to **MBN3** and 3’-TAMRA-labeled KRAS G4 | S16 |
| **Figure S23–67.** ^1^H NMR, ^13^C NMR and MS spectra of the final compounds | S16 |

**Table S1.** Oligonucleotides used in this study.

| Name | Sequence (5′→3′) | Structure in K^+^ Solution |
| --- | --- | --- |
| KRAS-22RT | AGGGCGGTGTGGGAATAGGGAA | Parallel G4 |
| KRAS-32R | AGGGCGGTGTGGGAAGAGGGAAGAGGGGGAGG | Parallel G4 |
| KRAS-28Rm | GGGAGGGAGGGAAGGAGGGAGGGAGGGA | Parallel G4 |
| KRAS RNA G4 | AAGGUGGCGGCGGCUCG | Parallel G4 |
| MYC | TGAGGGTGGGTAGGGTGGGTAA | Parallel G4 |
| c-Kit1 | AGGGAGGGCGCTGGGAGGAGGG | Parallel G4 |
| Bcl2 | GGGCGCGGGAGGAATTGGGCGGG | Parallel G4 |
| Tel24 | TTGGGTTAGGGTTAGGGTTAGGGA | Hybrid G4 |
| c-Kit* | GGCGAGGAGGGGCGTGGCCGGC | Antiparallel G4 |
| HRAS | TCGGGTTGCGGGCGCAGGGCACGGGCG | Antiparallel G4 |
| Bm | TAGGTTAGGTTAGGTTAGG | Antiparallel G4 |
| TBA | GGTTGGTGTGGTTGG | Antiparallel G4 |
| Hairpin | CGCGCGCGTTTTCGCGCGCG | Duplex DNA |
| duplex32 | CGCGCGCGCGCGCGTTTTCGCGCGCGCGCGCG | Duplex DNA |
| ctDNA | Calf Thymus DNA | Duplex DNA |
| FRET_KRAS | FAM-AGGGCGGTGTGGGAATAGGGAA-TAMRA | Parallel G4 |
| TAMRA_KRAS | 5'-TAMRA-KRAS-22RT or KRAS-22RT-TAMRA-3' | Parallel G4 |

**Table S2.** Primers used in this study.

| Gene | Forward primer (5′→3′) | Reverse primer (5′→3′) |
| --- | --- | --- |
| *KRAS* | ACACAAAACAGGCTCAGGACT | TGTCGGATCTCCCTCACCAA |
| *NRAS* | CCTATACAATGTATGTAATTTGTTTCC | CAATGCACCAAAGTTTTACAATATTTGAAC |
| *HRAS* | GCTGATCCAGAACCATTT | GTATCCAGGATGTCCAAC |
| *c-MYC* | GTGGCACCTCTTGAGGACCT | TGGTGCTCCATGAGGAGACA |
| *Bcl2* | GAGGATTGTGGCCTTCTTTG | GCCGGTTCAGGTACTCAGTC |
| *β-ACTIN* | GCATCCTGTCGGCAATGC | GTTGCTATCCAGGCTGTGC |

**Figure S1.** TO displacement assay of indolium-based analogs (**BN1**-**BN12**) and KRAS G4.

**Figure S2.** Absorption titration assay of indolium-based analogs (**BN1**-**BN12**) and KRAS G4.

**Figure S3.** Fluorescence quenching assay of indolium-based analogs (**BN1**-**BN12**) and 3’-TAMRA-labeled KRAS G4, with *K*_D_ values shown in the inset.

**Figure S4.** Growth inhibition of indolium-based analogs (**BN1**-**BN12**) on different types of tumor cells displayed by IC_50_ values, determined by CCK8 assay. The data were presented as mean ± SE (n=3).

**Figure S5.** Normalized CD spectra of the tested G4s in this study.

**Figure S6.** Competitive fluorescence recovery assay performed by increasing concentrations of different types of unlabeled DNAs added to 3’-TAMRA-labeled KRAS G4 mixed with **BN1**.

**Figure S7.** Absorption titration assay of **BN1** and different types of DNAs. The fitting of the titration data to the Benesi-Hildebrand equation was also listed.

**Figure S8.** Aromatic region in ^1^H NMR spectra of KRAS G4 in the absence and presence of different molar equivalents of **BN1**.

**Figure S9.** (A) Heatmap diagram of Pearson correlation coefficient between all samples in RNA sequencing assay. (B) Cluster analysis and heatmap diagram of the overview of the differentially expressed genes, where FDR < 0.05 and fold change > 2 or < 0.5. (C) GO categorization of biological process, cellular component and molecular function for the differentially expressed genes, where FDR < 0.05 and fold change > 2 or < 0.5.

**Figure S10.** Effects of Sotorasib on MAPK/ERK pathway in different types of tumor cells determined by western blot assay.

**Figure S11.** Effects of **BN1** on apoptosis-related proteins (A) and cell cycle-related proteins (B) in MDA-MB-231 cells determined by western blot assay.

**Figure S12.** (A) Representative images of MDA-MB-231 tumor spheroids stained with Calcein AM and PI. (B) Fluorescence intensity of Calcein AM and PI in tumor spheroids. The data were presented as mean ± SE (n=3). * P < 0.05, ** P < 0.01, *** P < 0.001 compared to the control group.

**Figure S13.** (A) Effects of **BN1** on the transcription of KRAS in 4T1 cells determined by RT-PCR assay. (B) Effects of **BN1** on the expression of MEK and ERK in 4T1 cells determined by western blot assay.

**Figure S14.** Effects of **BN1** on the induction of apoptosis (A) and cell cycle arrest (B) in 4T1 cells determined by Annexin V-PI double staining and PI staining, respectively.

**Figure S15.** Effects of **BN1** on apoptosis-related proteins (A) and cell cycle-related proteins (B) in 4T1 cells determined by western blot assay.

**Figure S16.** (A) Inhibition of migration in **BN1**-treated 4T1 cells determined by wound scrape assay. (B) Growth inhibition in **BN1**-treated 4T1 cells determined by colony formation assay. (C) Growth inhibition in **BN1**-treated 4T1 cells determined by tumor spheroid assay. The data were presented as mean ± SE (n=3). ** P < 0.01 compared to the control group.

**Figure S17.** Representative images of the excised tumors from all the groups in tumor implantation model.

**Figure S18.** (A) Body weights of the mice measured every other day until Day 25 after tumor implantation. (B) Weights of the major organs of the mice determined at the time of sacrifice. (C) Representative H&E staining images of the major organs of the mice. The data were presented as mean ± SE (n≥5).

**Figure S19.** Representative flow cytometric plots of CD4^+^ (A), CD8^+^ (B), CD4^+^ Foxp3^+^ (C), CD8^+^ IFNγ^+^ (D) T cells and PD-L1^+^ (E) cells, from both control group and 10 mg/kg **BN1** group.

**Figure S20.** Representative immunohistochemistry staining images for CD4, CD8, KRAS.

**Figure S21.** TO displacement assay of **MBN1** to **MBN3** and KRAS G4.

**Figure S22.** Fluorescence quenching assay of **MBN1** to **MBN3** and 3’-TAMRA-labeled KRAS G4, with *K*_D_ values shown in the inset.

**Figure S23.** ^1^H NMR spectrum of **BN1**

**Figure S24.** ^13^C NMR spectrum of **BN1**

**Figure S25.** MS spectrum of **BN1**

**Figure S26.** ^1^H NMR spectrum of **BN2**

**Figure S27.** ^13^C NMR spectrum of **BN2**

**Figure S28.** MS spectrum of **BN2**

**Figure S29.** ^1^H NMR spectrum of **BN3**

**Figure S30.** ^13^C NMR spectrum of **BN3**

**Figure S31.** MS spectrum of **BN3**

**Figure S32.** ^1^H NMR spectrum of **BN4**

**Figure S33.** ^13^C NMR spectrum of **BN4**

**Figure S34.** MS spectrum of **BN4**

**Figure S35.** ^1^H NMR spectrum of **BN5**

**Figure S36.** ^13^C NMR spectrum of **BN5**

**Figure S37.** MS spectrum of **BN5**

**Figure S38.** ^1^H NMR spectrum of **BN6**

**Figure S39.** ^13^C NMR spectrum of **BN6**

**Figure S40.** MS spectrum of **BN6**

**Figure S41.** ^1^H NMR spectrum of **BN7**

**Figure S42.** ^13^C NMR spectrum of **BN7**

**Figure S43.** MS spectrum of **BN7**

**Figure S44.** ^1^H NMR spectrum of **BN8**

**Figure S45.** ^13^C NMR spectrum of **BN8**

**Figure S46.** MS spectrum of **BN8**

**Figure S47.** ^1^H NMR spectrum of **BN9**

**Figure S48.** ^13^C NMR spectrum of **BN9**

**Figure S49.** MS spectrum of **BN9**

**Figure S50.** ^1^H NMR spectrum of **BN10**

**Figure S51.** ^13^C NMR spectrum of **BN10**

**Figure S52.** MS spectrum of **BN10**

**Figure S53.** ^1^H NMR spectrum of **BN11**

**Figure S54.** ^13^C NMR spectrum of **BN11**

**Figure S55.** MS spectrum of **BN11**

**Figure S56.** ^1^H NMR spectrum of **BN12**

**Figure S57.** ^13^C NMR spectrum of **BN12**

**Figure S58.** MS spectrum of **BN12**

**Figure S59.** ^1^H NMR spectrum of **MBN1**

**Figure S60.** ^13^C NMR spectrum of **MBN1**

**Figure S61.** MS spectrum of **MBN1**

**Figure S62.** ^1^H NMR spectrum of **MBN2**

**Figure S63.** ^13^C NMR spectrum of **MBN2**

**Figure S64.** MS spectrum of **MBN2**

**Figure S65.** ^1^H NMR spectrum of **MBN3**

**Figure S66.** ^13^C NMR spectrum of **MBN3**

**Figure S67.** MS spectrum of **MBN3**
